# Discovery of resident memory T cells in inflammatory vitiligo: A case report

**DOI:** 10.1097/MD.0000000000031007

**Published:** 2022-10-14

**Authors:** YanLi Xu, Bao-Xiang Zhang, Mao Lin, Lu Zhang

**Affiliations:** a School of Clinical Medicine, Weifang Medical University, Weifang, Shandong Province, P.R. China; b Department of Dermatology, Yidu Central Hospital, Weifang, Shandong Province, P.R. China; c Department of Dermatology, Chongqing Chinese Medicine Hospital, Yuzhong District, Chongqing, P.R. China.

**Keywords:** case report, CD8 positive T lymphocytes, inflammatory vitiligo, resident memory T cells, vitiligo

## Abstract

The purpose of this report was to describe resident memory cluster of differentiation 8 (CD8) + T cells may contribute to the progression of inflammatory vitiligo.

**Patient concerns:**

A 32-year-old male has a stable vitiligo for 1 year, then some patches present inflammatory erythema. Two years later, the inflammatory patches enlarged and joined together, and the remaining 2 common patches shows repigmentation and no change respectively. Both CD69 + CD8 + T cells and CD103 + CD8 + T cells showed marked increase in inflammatory vitiligo than common vitiligo.

**Diagnosis:**

Histological findings show that the numbers of lymphocytes are increased in inflammatory vitiligo than common vitiligo. Immunofluorescence staining show that the numbers of CD69 + CD8 + T cells demonstrated a marked increase in inflammatory vitiligo than common vitiligo.

**Interventions:**

Without any intervention.

**Outcomes:**

The previous upper 2 patches on the abdomen with erythematous rim were enlarged and joined together. However the lowest lesion with uninflamed common rim on the abdomen remained static, the one on the right groin showed spot-like repigmentation.

**Lessons:**

This case report demonstrates that resident memory CD8 + T cells may contribute to the progression of inflammatory vitiligo.

## 1. Introduction

Inflammatory vitiligo described as having a rim of raised erythema at the periphery of the depigmented patches is a rare subtype of vitiligo,^[1]^ and the etiology is poorly understood. Resident memory cluster of differentiation 8 (CD8) + T cells may be active players in disease maintenance. However, there is no clear relationship between inflammatory vitiligo and resident memory CD8 + T cells. We report a case of a 32-year-old male has a stable vitiligo for 1 year, then some patches present inflammatory erythema. Two years later, the inflammatory patches enlarged and joined together, and the remaining 2 common patches shows repigmentation and no change respectively. Both CD69 + CD8 + T cells and CD103 + CD8 + T cells showed marked increase in inflammatory vitiligo than common vitiligo.

## 2. Case report

**Figure 1.**
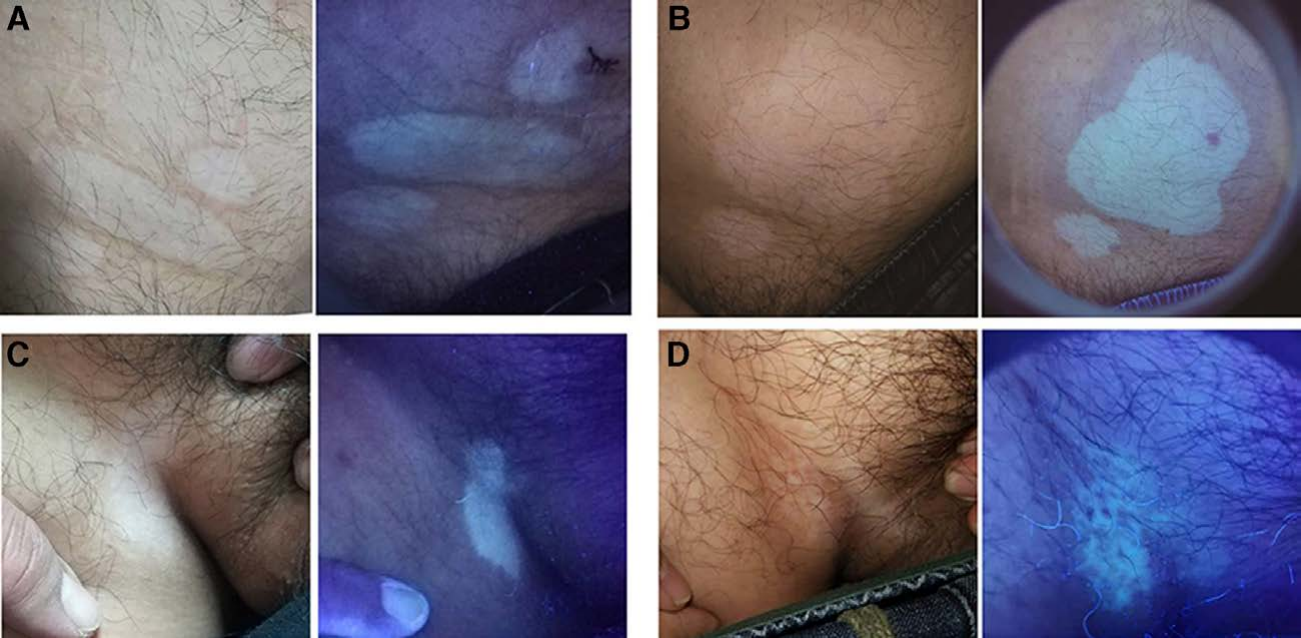
The white patches (a, b), and the changes two years later (c, d).

Two years ago, a 32-year-old Chinese male presented to our department of dermatology complaining of his white patches. Most patches remained unchanged for 1 year except 2 patches on the right abdomen which were found have a rim of erythema 1 day ago (Fig. 1a, b). All the lesions exerted a color of pale white under Wood’s lamp. Biopsies from the inflammatory erythematous rim of the top one and uninflamed common rim of the lowest one on abdomen were performed respectively. We clinically considered this case as inflammatory vitiligo, but the patient refused any treatment. Interestingly, 2 years later, the patient revisited our department complaining of the enlargement of the patches. When compared with previous data, the previous upper 2 patches on the abdomen with erythematous rim were enlarged and joined together. However the lowest lesion with uninflamed common rim on the abdomen remained static, and notably, the one on the right groin showed spot-like repigmentation (Fig. 1c, d).

**Figure 2.**
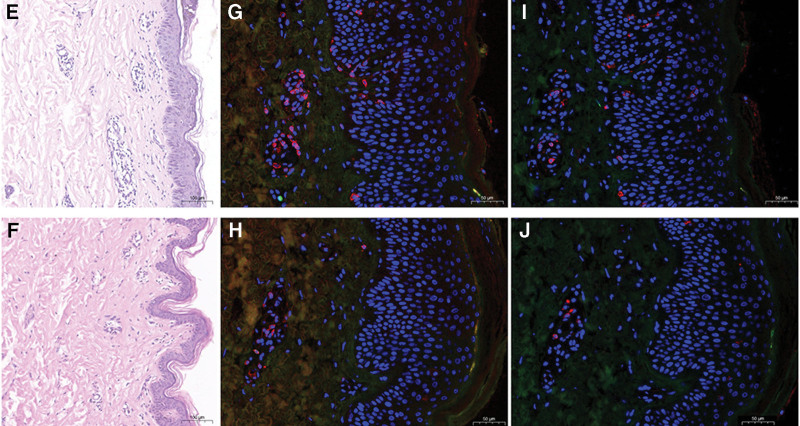
Histological findings from margenal lesion of inflammatory vitiligo (e) and common vitiligo (f), low magnification (hematoxylin and eosin, ×40); immunofluorescence staining (original magnification, ×200) (inflammatory vitiligo, common vitiligo) of CD69 + CD8 + T cells (g, h) and CD103 + CD8 + T cells (i, j). CD = cluster of differentiation.

Both the biopsies revealed mild spongiosis, interface dermatitis, focal basal vacuolization and subtle perivascular lymphocytic infiltration. However the numbers of lymphocytes are increased in inflammatory vitiligo than common vitiligo (Fig. 2e, f). The numbers of CD69 + CD8 + T cells demonstrated a marked increase in inflammatory vitiligo than common vitiligo (Fig. 2g, h), so as to the numbers of CD103 + CD8 + T cells (Fig. 2i, j).

## 3. Discussion

Inflammatory lesions is one of characterized clinical markers of active vitiligo besides Koebner phenomenon, trichrome lesions, inflammatory lesions and confetti-like depigmentation, and the inflammatory response may occur in the early stage of vitiligo.^[2]^ And the occurrence rates of this form of vitiligo can be estimated at 0.5% of all vitiligo cases.^[3]^ Different patches may be increased in size, decreased in size or stayed the same in 1 vitiligo patient.^[4]^ So inflammatory vitiligo may coexist with common vitiligo in clinical consequences, however the mechanism needs further study.

Recently the resident memory T (TRM) cells in vitiligo, one of the current research interest, is at the emerging stage.^[5,6]^ TRM cells marked by the expression of surface markers CD69 and CD103 are responsible for the recurrence of many autoimmune diseases.^[7]^ CD69 is the hallmark to define TRM in tissues through downregulation of surface expression of sphingosine 1 phosphate receptor 1. CD103, aka αE integrin, is another TRM marker.^[8]^ CD69 + CD103 + TRM and CD69 + CD103-TRM accumulate in the perilesional skin of vitiligo patients.^[9]^ Previous studies have shown CD8 + T cells are responsible for the destruction of melanocytes in vitiligo, and multiple groups identified CD8 + T cells possessing a TRM cell phenotype within vitiligo lesions.^[10]^ The proportion of CD69 + CD103 + CD8 + T cells accounted for 20% of CD8 + T cells in peripheral blood mononuclear cells, whereas 80% were found in the skin lesions of vitiligo.^[11]^ However, the expression of CD8 + TRM in inflammatory vitiligo has not been report. In this case, the numbers of both CD69 + CD8 + T cells and CD103 + CD8 + T cells are increased in the inflammatory vitiligo more than common vitiligo. Two years later, the patches of inflammatory vitiligo developed obviously. The results indicate that the CD8 + TRM cells in vitiligo may not be only related to relapse of vitiligo but also the progression of the disease. Under the stimulation of interleukin 15, the CD8 + TRM cells secrete perforin, granzyme-B and IFN-γ, which may induce melanocyte apoptosis directly.^[12]^ On the other side, CD8 + TRM cells produce chemokines C-X-C motif ligand 9 and C-X-C motif ligand 10 which bind to C-X-C-Motif Receptor 3 on the surface of recirculating memory T (TRCM) cells for the recruitment of TRCM to the skin.^[8]^

## 4. Conclusion

To our knowledge, this is the first report of an association between inflammatory vitiligo and CD8 + TRM cells. And it is interesting that there are 3 different outcomes in the neighboring three lesions in the same patient, however we had not an biopsy from the lesion on the right groin, this is an limitation of the paper. Further investigations are needed to unravel the underlying mechanism of TRM cells, which may be an effective therapeutic target.

## Author contributions

**Conceptualization:** BaoXiang Zhang,

**Data curation:** Lu Zhang,

**Writing – original draft:** YanLi Xu,

**Writing – review & editing:** Mao Lin, BaoXiang Zhang.
